# Supplementary material for: Robust COVID-19 Vaccine Responses Despite Filarial Co-Infection: Insights from a Lymphatic Filariasis Cohort in Ghana
Source: Vaccines (Basel). 2025 Mar 13;13(3):312. doi: 10.3390/vaccines13030312 (PMC11945955; doi:10.3390/vaccines13030312)
Supplement: Supplementary file 1 [file vaccines-13-00312-s001.zip › vaccines-3483597-supplementary.pdf]

Supplementary Materials

Figure S1. Comparable SARS-CoV-2 vaccination-induced IgA/IgG antibody response independent of lymphoedema stage in lymphatic filariasis individuals

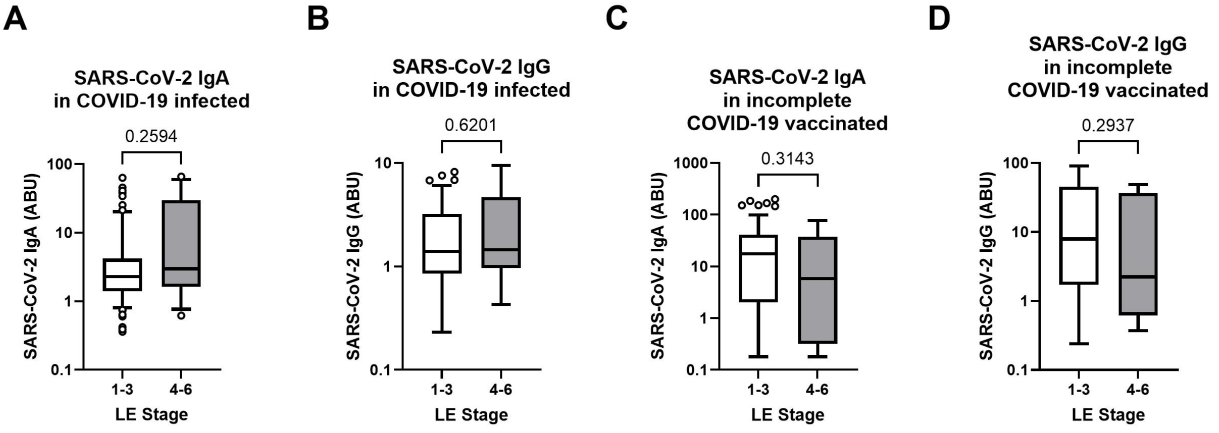

Figure S1. No effect of lymphoedema stage on infection- and vaccine-induced SARS-CoV-2 specific antibody response in lymphatic filariasis individuals. Comparable SARS-CoV-2 IgA and IgG antibody levels in COVID-19 infected (A+B) participants with lower (n=75) (white) lymphoedema stages compared to the group with increased pathology (n=12) (grey), as well as comparable levels among incomplete COVID-19 vaccinated (C+D) individuals with lower (n=55) and higher (n=8) LE pathology. Indicated p values were calculated using Mann-Whitney-U test. Bars represent the median ± IQR of antibody binding units (ABU). Significance is accepted if p <0.05.

Figure S2. No impact of circulating filarial antigen (CFA), ***Ascaris lumbricoides*** **or** ***Acanthocheilonema viteae*** **serostatus on SARS-CoV-2 specific IgG and IgA expression after COVID-19 vaccination**

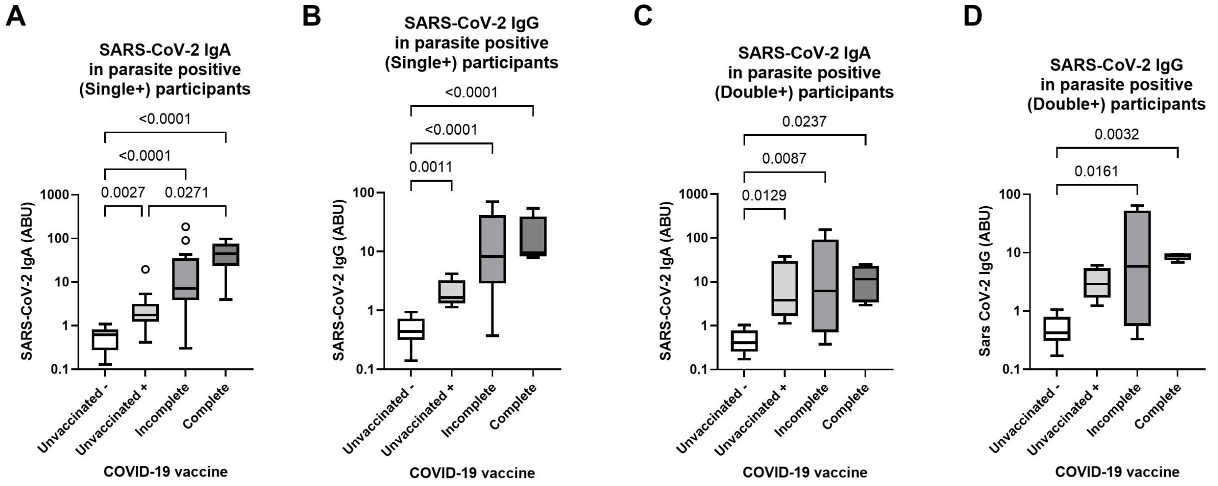

Figure S2. Robust SARS-CoV-2-specific antibody response in lymphatic filariasis patients after incomplete and complete COVID-19 vaccination, independent of the parasitic status. SARS-CoV-2 Spike specific IgA (A+C) and IgG (B+D) antibody response in incomplete (medium grey) and complete (dark grey) COVID-19 vaccinated lymphatic filariasis individuals independent of single (A+B) or double (C+D) seropositivity for Circulating filarial antigen (CFA), *Ascaris lumbricoides* and *Acanthocheilonema viteae* compared to the unvaccinated control group (white). Indicated p values were calculated using Kruskal-Wallis' test followed by Dunn's comparison post hoc to compare all groups. Bars represent the median ± IQR of antibody binding units (ABU). Significance is accepted if p <0.05

Figure S3. No effect of the parasitic status on the systemic cytokine and chemokine profile

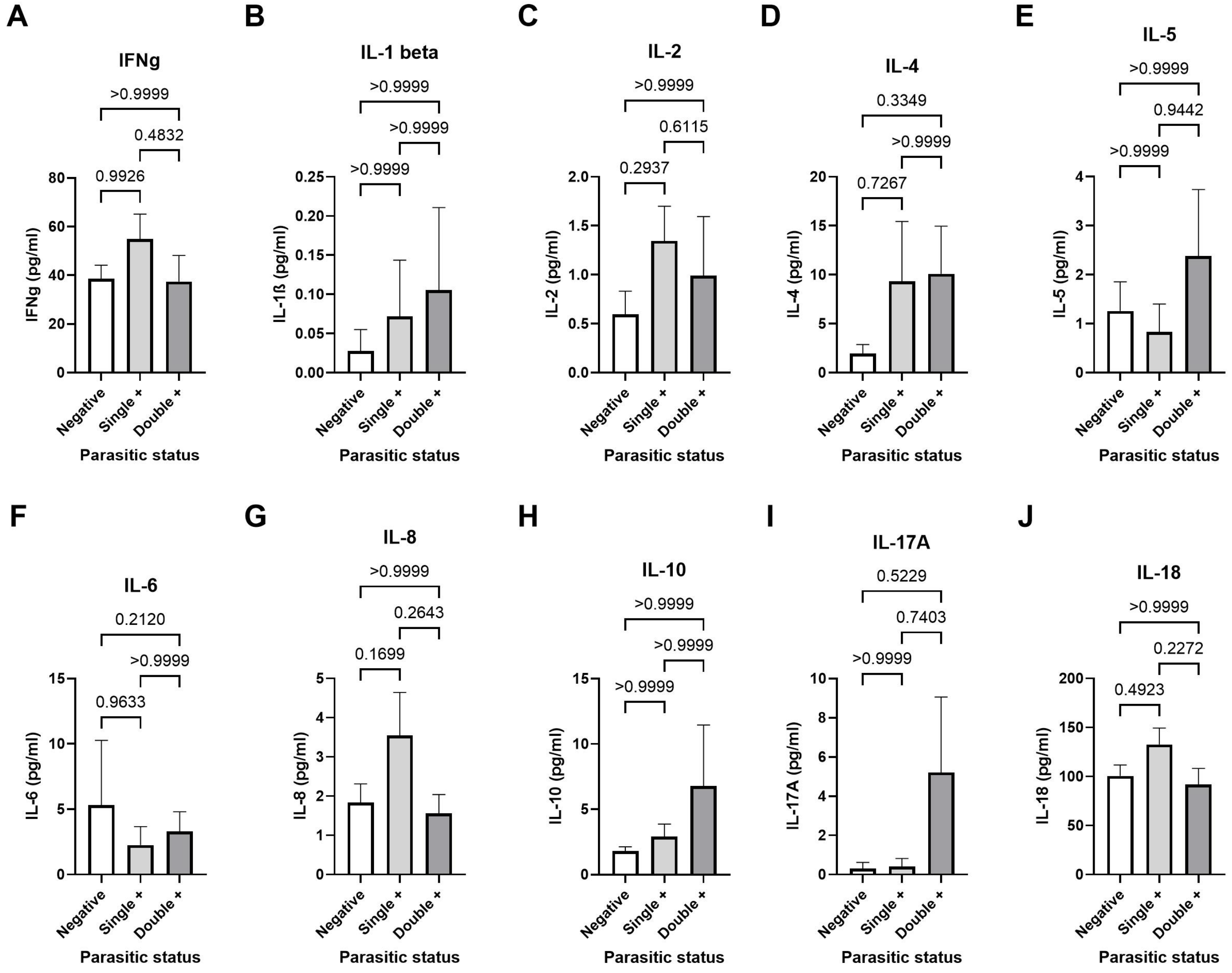

Figure S3. No effect of parasitic status on the systemic cytokine and chemokine profile of lymphatic filariasis individuals. Comparable levels of Th1, Th2, Th17 and Treg-related cytokines were detected within negative (white), single positive (light grey), and double positive (dark grey) participants, grouped according to three different filarial tests (Circulating filarial antigen, *Ascaris* IgG, A*canthocheilonema* IgG). Indicated p values were calculated using Kruskal-Wallis' test followed by Dunn's comparison post hoc to compare all groups. Bars represent the mean ± SEM of cytokines and chemokines (pg/ml). Significance is accepted if $p < 0.05$.

Figure S4. Comparable neutralizing potential of SARS-CoV-2 antibodies among SARS-CoV-2 Nucleocapsid (NCP) negative/positive incomplete and complete COVID-19 vaccinated lymphatic filariasis individuals

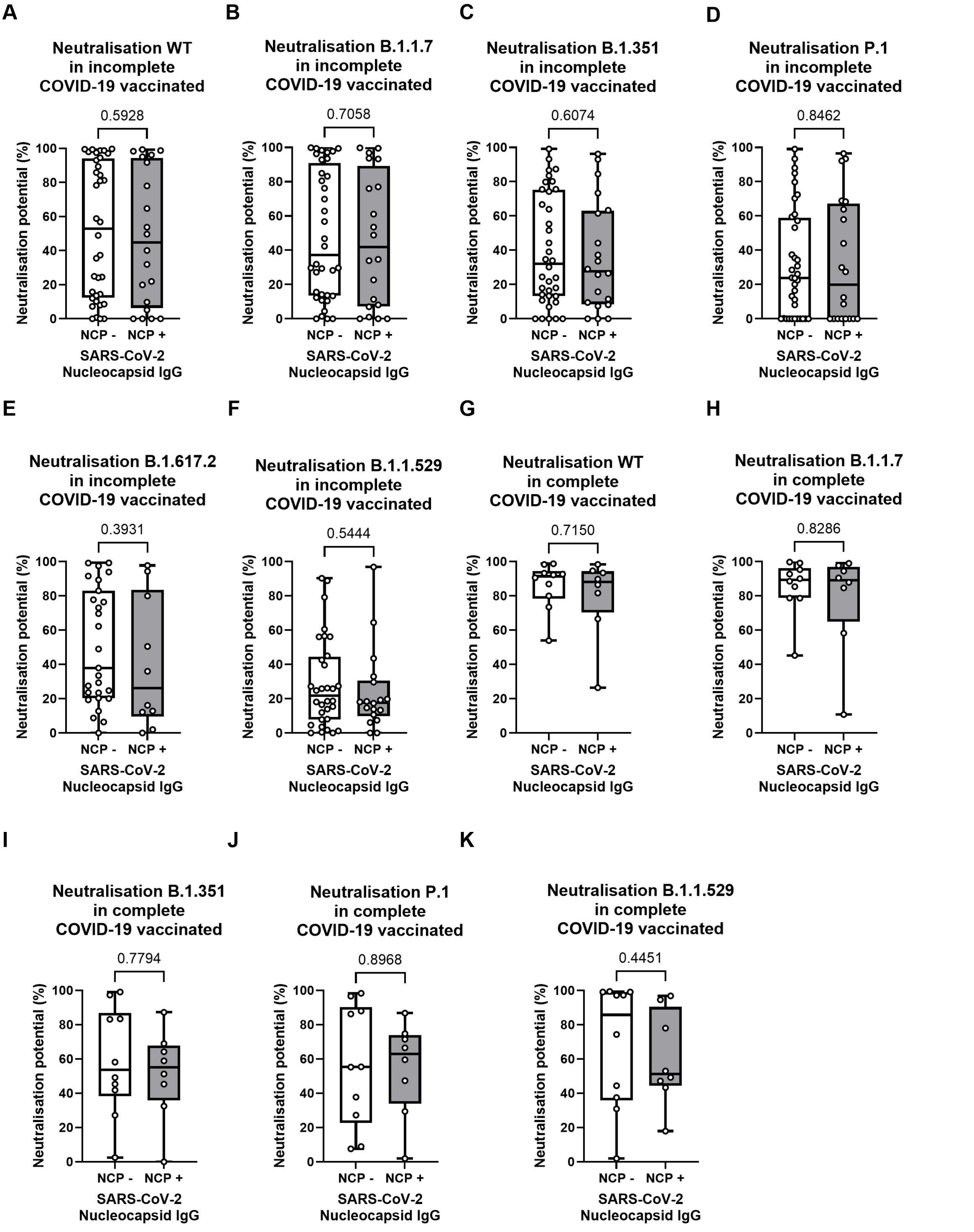

Figure S4. SARS-CoV-2 variant specific antibody neutralizing potential in anti-Nucleocapsid specific IgG negative and positive COVID-19 vaccinated lymphatic filariasis individuals. Comparable levels of SARS-CoV-2 neutralizing antibodies among SARS-CoV-2 Nucleocapsid (NCP) negative (white)/positive (grey) incomplete (A-F) and complete (G-K) COVID-19 vaccinated lymphatic filariasis individuals towards six different SARS-CoV-2 variants including: Wildtype (WT), Alpha (B.1.1.7), Beta (B.1.351), Gamma (P.1), Delta (B.1.617.2) and Omicron (B.1.1.529). Indicated p values were calculated using Mann-Whitney-U test. Bars represent the mean ± min.-max. of neutralizing potential (%) (A-K). Each dot represents an individual donor. Significance is accepted if $p < 0.05$.

S5. Comparable SARS-CoV-2 neutralizing capacity in incomplete and complete COVID-19 vaccinated lymphatic filariasis individuals towards six variants of concern (VoC) irrespective of parasitic status

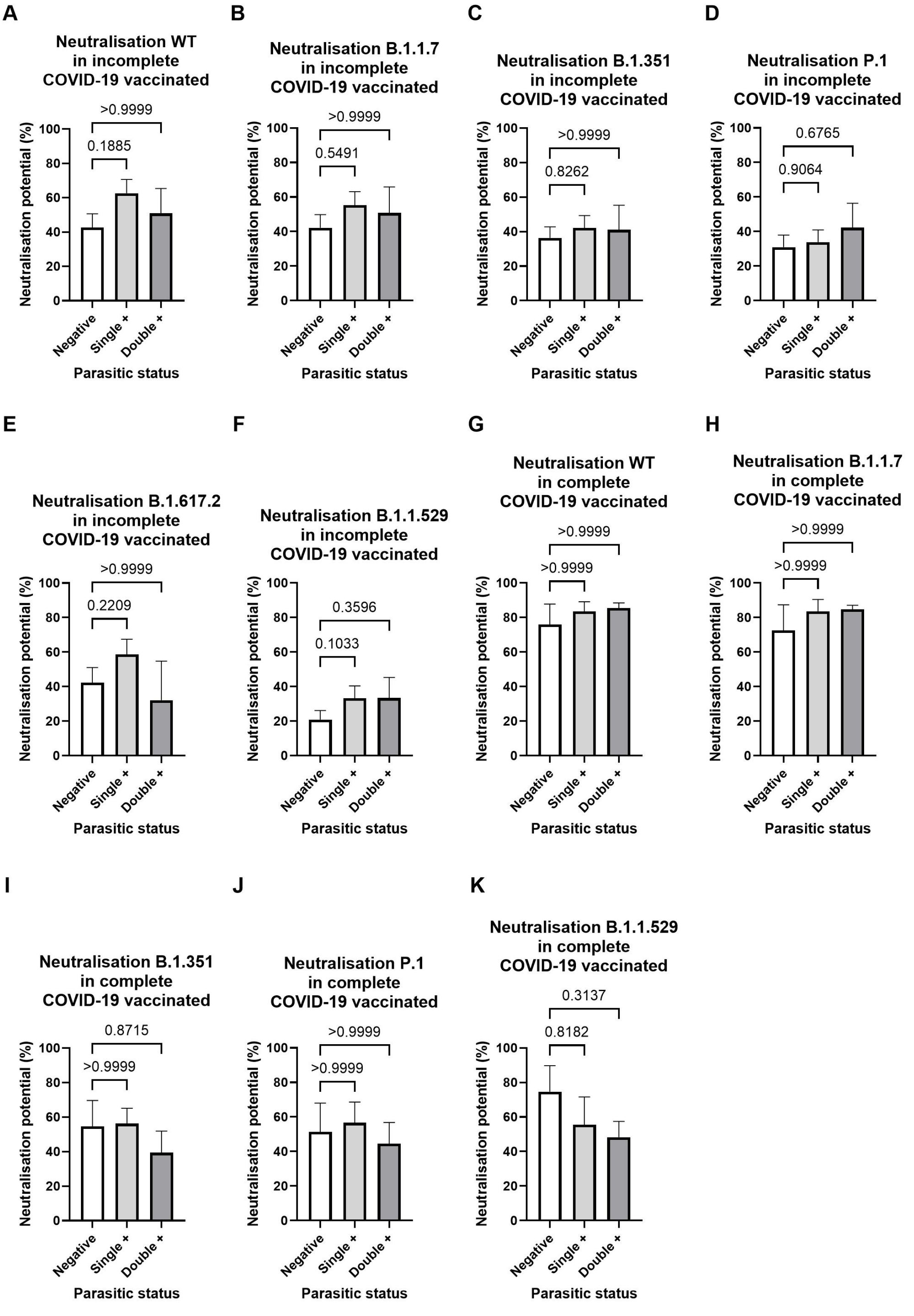

Figure S5. SARS-CoV-2 variant specific antibody neutralizing potential in COVID-19 vaccinated lymphatic filariasis individuals with a different parasitic status. High and comparable SARS-CoV-2 neutralizing antibodies in incomplete (A-F) and complete (G-H) COVID-19 vaccinated lymphatic filariasis individuals independent of single (n=17) (light grey) and double (n=9) (grey) seropositivity for Circulating filarial antigen (CFA), *Ascaris lumbricoides* IgG and *Acanthocheilonema viteae* IgG, towards six different SARS-CoV-2 variants including: Wildtype (WT), Alpha (B.1.1.7), Beta (B.1.351), Gamma (P.1), Delta (B.1.617.2) and Omicron (B.1.1.529) compared to the filarial negative control group (n=28) (white). Indicated p values were calculated using Kruskal-Wallis' test followed by Dunn's comparison post hoc to compare all groups. Bars represent the mean ± SEM of neutralizing potential (%). Significance is accepted if $p < 0.05$.
